# Quality of life in patients after vestibular schwannoma surgery

**DOI:** 10.1007/s00701-024-05936-z

**Published:** 2024-01-25

**Authors:** Jan Lazak, Jan Betka, Eduard Zverina, Ales Vlasak, Marketa Bonaventurova, Zuzana Balatkova, Martin Kana, Zdenek Fik

**Affiliations:** 1 Department of Otorhinolaryngology and Head and Neck Surgery, Charles University, First Faculty of Medicine, University Hospital Motol, Prague, Czech Republic; 2 Department of Neurosurgery for Children and Adults, Second Faculty of Medicine, University Hospital Motol, Prague, Czech Republic

**Keywords:** Quality of life, Vestibular schwannoma, Questionnaire, Surgery

## Abstract

**Aim:**

To evaluate the most important factors of quality of life in patients after vestibular schwannoma surgery.

**Materials and methods:**

Patients with unilateral sporadic occurrence of vestibular schwannoma who underwent surgery via suboccipital-retrosigmoid approach were included in the prospective study (2018–2021). Patients after previous Leksell gamma knife irradiation (or other methods of stereotactic radiosurgery) were excluded. Quality of life was assessed using 10 validated questionnaires that were distributed preoperatively, 3 months and 1 year after the surgery.

**Results:**

A total of 76 patients were included in the study, complete data were analysed in 43 of them (response rate 57%). Grade III and IV represented up to 70% of all tumors. Patients with larger tumors had a significantly higher risk of postoperative facial nerve paresis, liquorrhea and lower probability of hearing preservation. Patients with smaller tumors and those, who suffered from headaches before surgery had more frequent and severe headaches after surgery. Postoperative headaches were associated with higher incidence of anxiety and tinnitus. More frequent anxiety was also identified in patients with preoperative serviceable hearing who became deaf after surgery. Nevertheless, tinnitus and hearing impairment appeared to have less impact on overall quality of life compared to headaches and facial nerve function.

**Conclusion:**

According to our results, tumor size, postoperative function of the facial nerve and occurrence of postoperative headaches had the greatest influence on the overall postoperative quality of life in patients after vestibular schwannoma surgery.

## Introduction

Vestibular schwannoma (VS) is a benign tumor growing from the eighth cranial nerve. It is the most common infratentorial tumor with a histopathologically benign character that represents approximately 8% of all intracranial tumors [8]. A typical manifestation of VS includes a triad of symptoms – hearing impairment, tinnitus and vertigo; however, the spectrum of issues is wide and the severity of symptoms is specific to the individual [4]. Therapy management currently consists of 3 therapeutic modalities – surgical removal of the tumor (several different approaches), radiotherapy by stereotactic radiosurgery methods (multiple forms of radiation) or observation (wait and scan) [18]. Thanks to technological progress and the availability of imaging methods, the morbidity and mortality of patients treated for VS has dramatically decreased in recent decades. This fact has led to quality of life becoming the main criterion in the management of VS [16].

Despite the great progress in microsurgery techniques, surgical treatment of VS remains burdened by the risk of many complications, many of which may significantly impact the mentality, behavior and existing lifestyle of a patient [19]. An example would be a severe postoperative lesion of the facial nerve, which, apart from the risk of corneal damage due to insufficient lubrication and dysfunction of eyelid closure, is also associated with anxiety, depression and other behavioral disorders [2]. Also, post-surgery headaches may be so disabling for some patients that they significantly complicate or completely prevent their return to normal life and work processes [10]. Unilateral sensorineural hearing loss is usually one of the primary symptoms leading to the diagnosis in approximately 2/3 of patients with VS and at the same time one of the most frequent postoperative symptoms, which can lead to a deteriorated overall quality of life [22]. For the postoperative rehabilitation of unilateral hearing loss, patients today can be offered the CROSS/BiCROSS hearing aids (contralateral/bilateral routing of signals) as well as implantable systems using bone conduction (BAHD – bone anchored hearing devices) and, in indicated cases, a cochlear or brainstem neuroprosthesis [5]. Despite the good availability, effectiveness and safety of hearing compensation aids, a relatively small number of patients decide to use them [11]. The aim of this study was to identify the most important factors of quality of life after vestibular schwannoma surgery.

## Material and method

Between the years 2018 to 2021, a total of 127 patients diagnosed with vestibular schwannoma underwent surgery at the Department of Otorhinolaryngology and Head and Neck Surgery, 1st Faculty of Medicine of Charles University, University Hospital in Motol. Only patients with unilateral sporadic occurrence of vestibular schwannoma who were operated on using the retrosigmoid-suboccipital approach (RS) were included in the prospective questionnaire study. Patients who had previously undergone Leksell gamma knife radiotherapy (or other methods of stereotactic radiosurgery) were not included in the study. These criteria were met by a total of 108 patients (19 were excluded due to surgery via the translabyrinthine or combined approach). Seventy-six patients agreed to be included in the study. Complete results were obtained and assessed for 43 patients. Quality of life was assessed using 10 validated questionnaires that were always distributed to the patients before surgery, 3 months after surgery and 1 year after surgery.

- WHO Quality of Life-BREF (WHOQOL-BREF)
- Penn Acoustic Neuroma Quality of Life (PANQOL)
- Headache Disability Inventory (HDI)
- Dizziness Inventory (DI), (In-house Questionnaire)
- Hearing Handicap Inventory for Adults (HHIA)
- Tinnitus Handicap Inventory (THI)
- General Anxiety Disorder-7 (GAD-7)
- Zung Self-Rating Depression Scale (SDS)
- Facial Disability Index (FDI)
- Traditional Decisional Conflict Scale (DCS)

Age, gender, tumor size, hearing, facial nerve function, total duration of surgery, length of hospitalization and occurrence of postoperative complications were also assessed. The Koos grading scale was used to classify the size of the tumor. Hearing and facial nerve function were always assessed preoperatively as well as 3 months and 1 year after the surgery. To assess hearing, we used the American Academy of Otolaryngology–Head and Neck Surgery (AAO-HNS) guidelines for the evaluation of hearing preservation in acoustic neuroma, word recognition score (WRS) and pure tone average (PTA). Facial nerve function was assessed using the House-Brackmann (HB) classification.

### Surgical technique

All patients indicated for surgical therapy of vestibular schwannoma were operated on using the retrosigmoid-suboccipital approach. This surgical technique has already been described in our previous publication [9]. The surgeries were performed by a surgical team made up of a neurosurgeon and an otorhinolaryngologist. The function of the cranial nerves was monitored by a neurophysiologist.

Reconstructive surgery was performed on those patients whose facial nerve was transected. The technique and results are described in our previous publications [9].In patients with maintained serviceable hearing preoperatively, hearing was monitored using evoked brainstem response audiometry (ABR).

### Statistical analysis

The methods of descriptive and inductive statistics were used for the statistical analysis. Descriptive methods were used primarily for analysis of average, minimal and maximal value. We also used the Bravais-Pearson correlation coefficient. Inductive statistics were used for the analysis of dispersion and covariance. To test contrasts, we used Tukey HSD. The preconditions for use of a linear model were verified by the Shapiro–Wilk, Bartlett and Mauchly tests. The evaluation was performed at the statistical significance level *p* ≤ 0.05. Calculations were made in R Core Team 2022 (Vienna, Austria).

## Results

The results were statistically assessed in 43 of 76 patients who agreed to be included to the study (response rate = 57%). A total of 21 women and 22 men were indicated for surgery. The average age at the time of surgery was 47 years in men (25–70 years) and 49 years in women (26–66 years). Grade I and II tumors formed 30% (*n* = 13) of the cases; grade III and IV represented 70% (*n* = 30). The average length of hospitalization was 14 days (7–61 days); the average duration of the intracranial part of the surgery lasted 4.8 h (2.5–9.5 h).

**Fig. 1:**
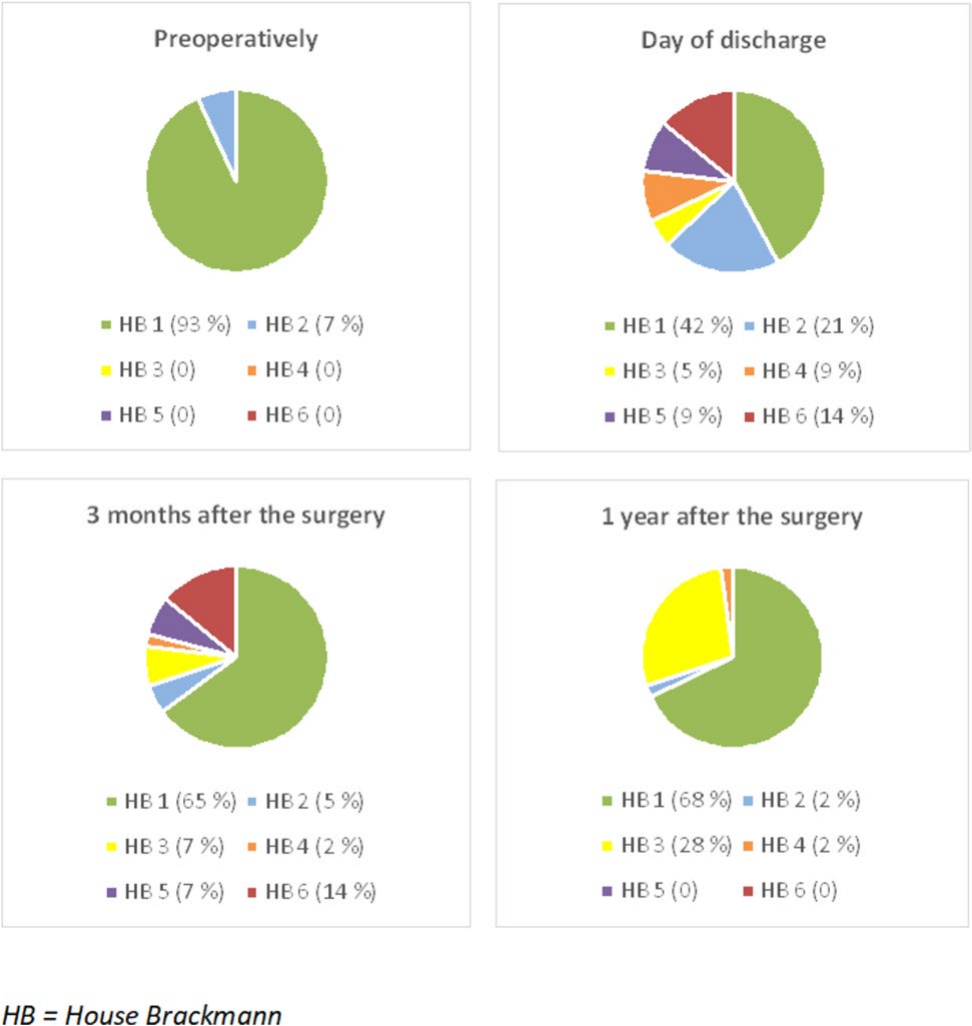
Facial nerve function – preoperatively, day of discharge, 3 month and 1 year after the surgery, (*n* = 43)

Preoperative and postoperative function of n.VII is described in Fig. 1. We found a statistically significant correlation between the size of the tumor and facial nerve function. Patients with grade I and II tumors reported better postoperative outcomes of n.VII function compared to patients with grade III and IV tumors (*p* = 0.002).

Due to intraoperative injury, n.VII was reconstructed in 12% of the patients (*n* = 5). Direct anastomosis was performed in 2 cases, one of which required the interposed sural nerve graft. Three patients were indicated for hemihypoglossal-facial (XII-VII) indirect anastomosis as modified by Darrouzet (68–109 days after the surgery). The function of n.VII improved in all patients who underwent the reconstruction, 80% of which (*n* = 4) achieved HB 3 (for synkinesis).

We further evaluated the questionnaire results based on the postoperative function of n.VII. Specifically, we compared the 3-month post-surgery results in patients with HB 1–3 and HB 4–6 (Table 1). One-year results were not assessed due to a small number of patients with n.VII function worse than HB 3 (*n* = 1). We have seen a statistically significant difference in the questionnaires:

- HDI (*p* < 0.001), HHIA (*p* = 0.017), GAD-7 (*p* < 0.001), SDS (*p* = 0.048), FDI (*p* = 0.03)

**Table 1:** Questionnaire score in patients with HB 1–3 (*n* = 33) and HB 4–6 (*n* = 10), 3 months after the surgery

		WHOQOL – BREF	PANQOL	HDI	DI	HHIA	THI	GAD-7	SDS	FDI	DCS
HB 1–3 (*n* = 33)	x	98.45	55.27	22.76	16.27	12	15.63	0.5	44.36	41.45	12.22
	CI	97.04–102.55	55.35–61.25	22.53–23.05	13.9–18.86	11.45–12.56	3.66–23.96	0.01–1.14	44.17–44.48	41.31–41.58	9.67–15.44
HB 4–6 (*n* = 10)	x	95.1	53.3	3.2	12.8	26.61	3	2.6	45.4	36.6	8.67
	CI	92.03–97.84	50.72–55.95	2.85–3.39	7.69–17.89	26–27.18	0.72–5.11	2.02–3.22	45.27–45.53	35.56–37.92	5.61–11.70
	*p*	> 0.05	> 0.05	< 0.001	> 0.05	0.017	> 0.05	< 0.001	0.048	0.03	> 0.05

*HB* House-Brackmann classification, *x* average questionnaire score, *CI* coincident interval 95%, *p* statistical significance level *p* ≤ 0.05

We can say that patients with worse postoperative function of n.VII suffered more often from anxiety and depression and achieved significantly worse results in FDI and HHIA questionnaire compared to patients with better n. VII outcomes.

According to AAO-HNS classification, before surgery 53% of the patients (*n* = 23) had serviceable hearing. In 17% of these patients (*n* = 4) the function of the cochlear nerve was preserved even after surgery. The average PTA and WRS value in patients with serviceable hearing postoperatively was 42 dB (33–49 dB) and 90% (90–100%). We proved a statistically significant correlation between the size of the tumor and hearing. Patients with grade I and II tumors had better audiometric results both before (*p* = 0.025) and after the surgery (*p* = 0.03) compared to patients with grade III and IV tumors.

We further evaluated the questionnaires results based on hearing. Specifically, we compared results of patients with serviceable hearing pre- and postoperatively with results from patients with nonserviceable hearing. A statistically significant difference was not seen in any of the questionnaires. We also compared questionnaire data in patients with postoperative hearing deterioration from serviceable to nonserviceable level with a group of patients whose hearing was assessed as nonserviceable even before the surgery (Table 2). A statistically significant difference was seen in the questionnaire:

- GAD-7 (after 3 months: *p* = 0.006, after 1 year: *p* < 0.001)

**Table 2:** Questionnaire score in patients with postoperative hearing deterioration from serviceable to nonserviceable level (Group A) and patients with nonserviceable hearing even before the surgery (Group B)

		WHOQOL -BREF	PANQOL	HDI	DI	HHIA	THI	GAD-7	SDS	FDI	DCS
Preoperatively Group A (*n* = 23)	x	102.1	43.82	5.5	12.45	13.37	18.53	4.35	46.1	44.05	9.89
	CI	92.66–105.05	35.77–50.72	4.99–6.12	7.53–19.76	8.84–17.48	17.7–19.5	3.9–4.99	45.66–46.76	43.17–45.3	8.44–11.1
Preoperatively Group B, (*n* = 20)	x	104.1	46.05	12,7	12.1	14.11	13.3	2.55	45.45	41.55	6.35
	CI	99.88–107.33	35.13–49.69	12.27–13.36	5.54–17.83	10.09–18.15	12.71–14.47	2.03–3.23	44.99–45.94	38.28–44.5	2.26–11.02
3 months after Group A (*n* = 23)	x	95.75	54.8	22.95	16.35	28.4	16	3.05	44.15	40.35	6.47
	CI	90.23–103.8	41.22–68.58	19.98–25.5	11.99–22.06	24.76 -32.05	15.13–16.87	2.94–3.22	42.11–44.27	39.54–41.7	5.62–7.36
3 months after Group B (*n* = 20)	x	97.77	55.26	15.72	15.49	24.46	13.13	2.33	44.69	40.21	11.32
	CI	91.54–102.3	49.01–61.14	12.62–18.19	9.72–20.7	20.14–28.33	12.28–14.07	2.2–2.47	43.51–45.42	39.24–41.53	4.86–17.51
	*p*	> 0.05	> 0.05	> 0.05	> 0.05	> 0.05	> 0.05	0.006	> 0.05	> 0.05	> 0.05
1 year after Group A (*n* = 23)	x	98.53	56.95	27.89	16.05	31.5	20	2.95	42.9	42	10.3
	CI	94.26–104.75	51.35–64.21	21.62 -32.85	7.4–18.31	26.8–35	19.03–20.79	2.8–3.07	41.77–44.07	40.78–42.94	9.49–11.23
1 year after Group B (*n* = 20)	x	99.32	55.23	18.95	14.88	28.56	16.74	2.74	43.56	40.95	10.85
	CI	92.86–103.91	54.39–66.01	13.53–24.83	14.02–15.78	24.87–32.73	15.9–17.67	2.58–2.87	42.62–44.72	39.85–42.05	10.19–11.96
	*p*	> 0.05	> 0.05	> 0.05	> 0.05	> 0.05	> 0.05	< 0.001	> 0.05	> 0.05	> 0.05

*x* average questionnaire score, *CI* coincident interval 95%, *p* statistical significance level *p* ≤ 0.05

Patients with new onset of postoperative hearing loss were more prone to anxiety compared to patients who already had nonserviceable hearing before the surgery.

A total of 72% (*n* = 31) of patients reported having tinnitus before the surgery, 53% (*n* = 23) after 3 months and 65% (*n* = 28) 1 year after surgery.

Tinnitus subsided immediately after the surgery for 47% of the patients (*n* = 11); however, it occurred again within 3 to 12 months in more than half (55%) of the patients. Tinnitus subsided permanently in 45% of the patients (*n* = 5), of which 80% (*n* = 4) were patients who had the cochlear nerve transected during surgery. In one case tinnitus subsided permanently while serviceable hearing has been maintained.

Patients who preoperatively did not have tinnitus and concurrently had serviceable hearing represented 12% (*n* = 5) of the patients studied. Those without tinnitus but with nonserviceable hearing preoperatively represented 14% (*n* = 6). Half of the patients whose cochlear nerve function was preserved developed new tinnitus after surgery (*n* = 2). There was only 1 patient with a new onset of tinnitus and concurrent nonserviceable hearing postoperatively.

We also assessed questionnaire results based on the presence of tinnitus. Specifically, we assessed results in patients who had been tinnitus free before the surgery and in whom tinnitus occurred after surgery. A statistically significant difference was not seen in any of the questionnaires.

More than 40% of patients (*n* = 18 cases) reported headaches before surgery, 60% patients 3 months after surgery (*n* = 26) and 63% of them 1 year after surgery (*n* = 27). With regard to gender, headaches both before and after surgery most frequently occurred in women (61% preoperatively, 58% 3 months and 59% 1 year after the surgery). Patients who already suffered from headaches before surgery had more frequent and worse pain after surgery (after 3 months: *p* < 0.001, after 1 year: *p* < 0.001). We also found a statistically significant correlation between headaches and size of tumor. Patients with smaller tumors also reported greater headaches after the surgery (after 3 months: *p* = 0.004, after 1 year: *p* = 0.019).

We also analyzed results from questionnaires of patients with headaches (preoperative, 3 months and 1 year after the surgery) and patients who did not report headaches (preoperative, 3 months and 1 year after the surgery), (Table 3). A statistically significant difference was seen in the case of the questionnaires:

- GAD-7 (before surgery: *p* = 0.035, after 1 year: *p* = 0.042), THI (before surgery: *p* = 0.02, after 1 year: *p* = 0.04)

**Table 3:** Questionnaire score in patients with and without headaches preoperatively, 3 months and 1 year after the surgery

		WHOQOL – BREF	PANQOL	HDI	DI	HHIA	THI	GAD-7	SDS	FDI	DCS
Preoperatively with headaches (*n* = 18)	x	103.5	46.4	19	13	14.1	21	4.7	47	43.7	9
	CI	101.38–104.57	42.79 -52.01	15.77–21.06	9.69–15.02	11.51–15.51	18.02–23.40	3.97–5.25	35.99–55.79	34.7–47.79	1.64–14.92
Preoperatively without headaches (*n* = 25)	x	104.7	42	0	11.5	12.9	11	2	45	43	6
	CI	99.66–105.28	33.73–48.92	0	9.96–13.36	13.44–17.45	8.19–14.21	1.49–2.67	25.91–49.88	37.59–58.96	0.46–15.16
	*p*	> 0.05	> 0.05	> 0.05	> 0.05	> 0.05	0.02	0.035	> 0.05	> 0.05	> 0.05
3 months with headaches (*n* = 26)	x	94.1	58.7	30.1	16.5	28.9	19.2	2.7	44.5	38.7	14.2
	CI	90.31–95.07	50.16–66.70	28.18–32.34	13.76–19.75	24.20–37.70	18.08–20.27	2.10–3.23	35.84 -62.14	29.08–49.27	5.83–16.20
3 months without headaches (*n* = 17)	x	103.1	48.8	0	13.8	14.5	2.8	1.3	44.7	42.2	7
	CI	100.64–104.98	40.58–61.37	0	11.57–13.97	9.63–24.38	1.82–3.92	0.77–1.91	33.16–53.01	42.12–64.11	4.9–15.3
	*p*	> 0.05	> 0.05	> 0.05	> 0.05	> 0.05	> 0.05	> 0.05	> 0.05	> 0.05	> 0.05
1 year with headaches (*n* = 27)	x	106	48.3	19.5	13	19.3	15.3	1.7	42.3	41	9.6
	CI	104.02–108.02	47.53–51.55	17.47–23.35	11.13–13.97	7.07–30.13	14.5–16.65	1.17–2.03	32.84–60.21	37.93–57.4	3.25–16.19
1 year without headaches (*n* = 16)	x	105	52	0	13.75	7	5	1.4	35	41.5	7.25
	CI	104.02–107.89	39.38–64.74	0	11.82–14.57	1.17–9.09	3.92–6.13	0.86–1.99	28.85–55.59	38.02–55.32	1.05 -14.21
	*p*	> 0.05	> 0.05	> 0.05	> 0.05	> 0.05	0.04	0.042	> 0.05	> 0.05	> 0.05

*x* average questionnaire score, *CI* coincident interval 95%, *p* statistical significance level *p* ≤ 0.05

Patients with pre- and postoperative headaches were more prone to anxiety and had more frequent tinnitus than patients without headaches.

Postoperative liquorrhea manifested in 30% of patients. In 25% (*n* = 11) as a cerebrospinal fluid pseudocyst, 3 cases of which also manifested with rhinoliquorrhea. Separate rhinoliquorrhea occurred in 5% of them (*n* = 2). Out of all cases, the occurrence of postoperative liquorrhea was more common in patients with grade III and IV tumors (*p* < 0.001). Postoperative liquorrhea was successfully managed with puncture and application of fibrin glue epidurally in 69% (*n* = 9) of patients, 31% of patients (*n* = 4) required surgical intervention under general anesthesia. In one case, the postoperative course was complicated by the development of acute meningitis.

We assessed the results of questionnaires in patients with postoperative complications and without, (Table 4). A statistically significant difference was seen only in patients with cerebrospinal fluid pseudocyst in the questionnaire:

- HDI (after 3 months: *p* = 0.03, after 1 year: *p* = 0.02)

**Table 4:** Questionnaire score in patients with and without postoperative CSF pseudocyst

	WHOQOL – BREF		PANQOL	HDI	DI	HHIA	THI	GAD–7	SDS	FDI	DCS
3 months after with CSF pseudocyst (*n* = 11)	x	96.18	57.09	19.82	14.54	21.09	17.45	1.82	42.73	40.27	13.2
	CI	95.65–99.15	54.02–60.16	18.21–21.43	12.59–17.1	20.27–23.48	16.49–19.78	0.93–2.40	40.68–44.04	37.05–42.85	12.26–15.23
3 months after without CSF (*n* = 32)	x	98.19	54.03	17.66	15.78	23.94	11.06	2.22	45.25	40.24	10.87
	CI	94.57–100.17	52.12–55.94	16.84–18.48	13.71 – 19.08	22.85–27.92	7.85–14.10	0.99–3.40	44.82–49.11	36.84 –43.09	6.76–12.02
	*p*	> 0.05	> 0.05	0.03	> 0.05	> 0.05	> 0.05	> 0.05	> 0.05	> 0.05	> 0.05
1 year after with CSF (*n* = 11)	x	94.6	53	24.73	13.55	21.45	20.4	3.45	43.82	42.27	9.18
	CI	93.24–97.89	49.11–56.89	22.06–27.04	12.87 –16.74	18.96 –21.65	18.04–22.2	1.08–3.98	42.81–46.56	39.43–42.88	6.79–11.89
1 year after without CSF (*n* = 32)	x	98	53.06	19.48	14.56	27.25	14.5	2.03	43.22	41.52	11.31
	CI	95.23–98.07	56.92–57.89	19.01–19.95	10.47–14.26	24.86–30.77	13.69–18.03	1.60–4.37	40.04–44.13	39.56 –43.3	7.2–12.64
	*p*	> 0.05	> 0.05	0.02	> 0.05	> 0.05	> 0.05	> 0.05	> 0.05	> 0.05	> 0.05

*x* average questionnaire score, *CI* coincident interval 95%, *p* statistical significance level *p* ≤ 0.05

Patients whose postoperative period was complicated by the development of a cerebrospinal fluid pseudocyst experienced more frequent and severe headaches 3 months and 1 year after surgery.

## Discussion

The ability to voluntarily control facial expressions, contingent on the appropriate function of the facial nerve, is a crucial element of human communication and social interaction [9]. Any dysfunction of n.VII, therefore, has a substantial impact on the psychological state and behavior of a patient [2, 19] In agreement with literature we noted a greater occurrence of depression and anxiety disorders in patients with severe lesions of n.VII than in patients with good postoperative function of n.VII [14]. The occurrence of more frequent and severe headaches in the group of patients with better postoperative n. VII function can be explained by the fact that postoperative headaches are more common in patients with smaller tumors [28, 29]. At the same time, patients with smaller tumors report better outcomes of postoperative n. VII function [6]. Better results of the HHIA questionnaire in patients with better postoperative facial nerve function may be also related to smaller tumor size [23].

The success rate of hearing preservation ranges between 10 and 100% and, as in our case, one of the most important predictive factors for hearing preservation is the size of the tumor [13, 15, 23]. Patients with unilateral deafness usually complain of impaired speech discrimination in loud environments and a limited ability to recognize the direction of incoming sound as a result of mononeural hearing and the acoustic (head) shadow [5]. However, less than 40% of all patients who could otherwise benefit from rehabilitation opt for compensatory hearing aids [27, 32]. These results may be interpreted either that patients are not informed well enough about the current options for hearing rehabilitation, or that severe postoperative hearing impairment is not a significant enough factor, with regard to overall quality of life, to motivate patients to seek further treatment. The idea that unilateral hearing impairment may not, according to our results, be necessarily associated with a significant decrease in quality of life has been mentioned also by Pruijn et al. [11, 27]. In contrast, according to Tveiten et. al. patients with bilateral good hearing (AA) according to the American Academy of Otolaryngology-Head and Neck Surgery classification scored significantly better than patients with unilateral hearing loss (AD) on a hearing inventory and a disease-specific QoL instrument [32].

Tinnitus occurs in approximately 50 to 80% of VS patients before surgery; in our case it was 72% [24, 31]. Various results can be found in the literature regarding tinnitus and hearing preservation [12, 17]. According to Chovanec et al., the most risky group in terms of persistence or onset of tinnitus are patients whose auditory nerve was preserved yet who still suffered hearing loss after the surgery [13]. Similar to Pruijn et al. or Carlson et. al., we did not identify a statistically significant correlation between the quality of life and tinnitus [11, 27]. However, a limitation of our results may be the relatively low number of respondents who developed new tinnitus after surgery.

The incidence of POH ranges between 0 and 73% depending on the literature [4]. As in our study, younger age, female gender and smaller tumor size are considered negative prognostic factors [28, 29]. More frequent occurrence of POH in patients with smaller tumors can be explained in some cases, according to Aihara et al., by the patient’s insufficient ability to reflect on indication for surgery even in the case of small and often asymptomatic tumors. This may lead to the onset of psychological stress and ultimately also increased risk of POH [1]. As in our study, Levo confirm a significantly higher incidence and severity of POH in patients who already suffered from headaches before the surgery [20]. Compared to Carlson et al., Nicoucar et al. and others who showed significantly worse results in the PANQOL questionnaire of POH patients, we did not find a statistically significant correlation between the quality of life and POH [3, 11, 25]. Even so, it is noticeable that patients with POH had worse results in the PANQOL and QoL questionnaires compared to patients without POH. Rimaaja et al. also identified more patients prone to depression in the group of patients with POH. In our study, we found a more frequent occurrence of anxiety and also tinnitus in the patients with POH [28]. However, considering the relatively low number of respondents, the higher incidence of tinnitus in patients with POH may be a coincidental finding.

Postoperative liquorrhea occurred in 30% of our patients, which corresponds with the incidence stated in literature [4, 30]. As in the study by Brennan et al., we identified tumor size as a significant predictive factor of postoperative liquorrhea [7]. Postoperative liquorrhea is, according to Pross et al., often accompanied by headaches and alteration of the patient’s mental state [26]. In our work, we proved a significantly higher incidence of headaches in patients with postoperative liquorrhea pseudocysts but in contrast to the literature, these were long-term postoperative headaches, not headaches as a common early symptom of postoperative liquorrhea [21, 26]. It is interesting, that even in a situation where the postoperative liquorrhea has long been resolved, these patients show a higher incidence of long-term headaches compared to patients in whom no liquorrhea was detected. The effect of surgical interventions or stigmatization and physical and emotional stress as a known trigger cause of postoperative headaches may be considered [20, 28].Although most of our findings agree with the literature, some new findings will require further detailed analysis. The reason is the relatively low number of respondents included in the prospective study, the relatively low response rate and the risk of coincidental findings. It can be explained by the large number of questionnaires and the time requirement and decreasing motivation to complete questionnaires over time. A number of patients also had to be excluded for not complying with the required check-up interval due to the ongoing covid-19 pandemic.

## Conclusion

Tumor size has been shown to be one of the most significant predictors of quality of life in patients after vestibular schwannoma surgery. Larger tumors were associated with a higher risk of facial nerve paresis development, occurrence of postoperative liquorrhea and lower probability of hearing preservation. Conversely, patients with smaller tumors suffered more often from severe and frequent headaches. At the same time, postoperative headaches were associated with higher incidence of anxiety and tinnitus. More frequent anxiety was also identified in patients with preoperative serviceable hearing who became deaf after surgery. In patients with tinnitus, we found no statistically significant correlation with questionnaires assessing the quality of life. According to our results, postoperative facial nerve function and headache have been shown to be some of the most significant factors affecting overall quality of life. However, these findings will require further detailed analysis on a larger group of patients due to the risk of coincidental findings in the relatively small cohort of respondents.
